# Serum TLR4, NF-κB, and NLRP3 Levels in Children with Attention-Deficit/Hyperactivity Disorder: A Case–Control Study

**DOI:** 10.3390/children13091278

**Published:** 2026-09-21

**Authors:** Esra Hoşoğlu, Züleyha Ulusoy, Nihal Turkmen Alemdar, Yüksek Aliyazıcıoğlu, Volkan Şahin, Bahadır Turan

**Affiliations:** 1 Department of Child and Adolescent Psychiatry, Faculty of Medicine, Karadeniz Technical University, 61080 Trabzon, Türkiye; 2 Department of Child and Adolescent Psychiatry, Ministry of Health, Siverek State Hospital, 63600 Şanlıurfa, Türkiye; 3 Department of Medical Services and Techniques, Vocational School of Health Services, Recep Tayyip Erdoğan University, 53100 Rize, Türkiye; 4 Department of Medical Biochemistry, Faculty of Medicine, Karadeniz Technical University, 61080 Trabzon, Türkiye

**Keywords:** ADHD, neuroinflammation, NF-κB, TLR4, NLRP3

## Abstract

**Highlights:**

**What are the main findings?**

• Serum NF-κB and NLRP3 levels were significantly higher in children with ADHD than in healthy controls, whereas serum TLR4 levels did not differ between groups.
• Serum NF-κB levels were positively associated with teacher-rated hyperactivity, while no consistent associations were observed for TLR4 or NLRP3.

**What are the implications of the main findings?**

• The findings provide preliminary evidence of alterations in peripheral NF-κB and NLRP3 levels in children with ADHD.
• The differing findings for TLR4, NF-κB, and NLRP3 highlight the complexity of peripheral inflammatory alterations in ADHD and warrant further investigation in larger, longitudinal studies.

**Abstract:**

**Background:** Increasing evidence suggests that inflammatory processes may be involved in the pathophysiology of attention-deficit/hyperactivity disorder (ADHD). However, clinical data regarding TLR4, NF-κB and NLRP3 in children with ADHD remain limited. This study aimed to compare serum TLR4, NF-κB, and NLRP3 levels between children with ADHD and healthy controls and to examine the associations between these inflammatory markers and ADHD symptom severity. **Methods**: A total of 42 children with ADHD and 40 age- and sex-matched healthy controls were enrolled. ADHD symptoms were assessed using the Conners’ Parent Rating Scale–Revised Short Form (CPRS-RS) and the Conners’ Teacher Rating Scale–Revised Short Form (CTRS-RS). Serum TLR4, NF-κB, and NLRP3 levels were measured using enzyme-linked immunosorbent assay (ELISA). Between-group comparisons were performed after adjustment for age, sex, and body mass index (BMI), and correlations between inflammatory markers and symptom severity were evaluated. **Results:** Serum NF-κB and NLRP3 levels were significantly higher in children with ADHD than in healthy controls, whereas serum TLR4 levels did not differ between groups. Serum NF-κB levels showed a moderate positive correlation with teacher-rated hyperactivity scores, while no significant associations were observed between TLR4 or NLRP3 levels and ADHD symptom severity in the ADHD group. **Conclusions:** Our findings provide preliminary clinical evidence of altered NF-κB and NLRP3 levels in children with ADHD. The absence of a significant difference in serum TLR4 levels, despite increased NF-κB and NLRP3 levels, suggests that these inflammatory components may be differentially altered in ADHD. Further longitudinal and mechanistic studies are needed to clarify the relevance of these inflammatory alterations to ADHD pathophysiology.

## 1. Introduction

Attention-deficit/hyperactivity disorder (ADHD) is a neurodevelopmental disorder involving developmentally inappropriate levels of inattention, hyperactivity, and impulsivity that interfere with academic, social, and everyday functioning [1]. It affects approximately 5% of children worldwide, making it one of the most prevalent neurodevelopmental disorders in childhood [2,3]. Beyond its high prevalence, ADHD is linked to a range of long-term adverse outcomes, including poor educational and occupational achievement, psychiatric comorbidities, substance use disorders, injuries, and considerable socioeconomic burden [2]. Although the etiology of ADHD remains incompletely understood, accumulating evidence suggests that its pathophysiology involves genetic, environmental, and immunological factors [3,4].

Neuroinflammation has emerged as a potential mechanism linking these factors to ADHD. Several prenatal and early-life exposures associated with an increased risk of ADHD have also been linked to activation of inflammatory pathways during critical stages of neurodevelopment [4]. In addition, epidemiological studies have reported an increased prevalence of immune-related disorders among individuals with ADHD, while clinical studies have demonstrated altered circulating inflammatory biomarkers, although the findings have been inconsistent [2,4,5]. Together, these findings support a possible role of inflammation in ADHD.

The TLR4/NF-κB/NLRP3 signaling pathway has emerged as a potential molecular mechanism underlying neuroinflammation [6,7]. As a member of the Toll-like receptor family, TLR4 functions as a pattern-recognition receptor (PRR), enabling the innate immune cells to recognize molecular signatures associated with pathogens and tissue damage. In the central nervous system, TLR4 regulates neuroimmune responses and, upon activation, signals through MyD88-dependent and MyD88-independent pathways to activate NF-κB and other downstream inflammatory mediators [8,9]. Clinical findings across several psychiatric conditions point to a role for TLR4 signaling in their pathophysiology. Patients with schizophrenia show elevated TLR4 protein expression in the prefrontal cortex, while individuals with major depressive disorder exhibit higher peripheral TLR4 expression than healthy controls [10,11,12,13]. Similarly, children with autism spectrum disorder (ASD) have been reported to show enhanced TLR4/NF-κB signaling in B cells and higher circulating soluble TLR4 levels than healthy peers [14,15].

NF-κB refers to a family of transcription factors that play a central regulatory role in immune and inflammatory signaling [16]. In the central nervous system, NF-κB is widely expressed in neurons and glial cells and, as a major downstream effector of TLR4 signaling, regulates the transcription of a broad range of inflammatory mediators [9,17]. Increased NF-κB activation has been reported in several psychiatric disorders. Elevated NF-κB expression has been demonstrated in the serum of children with ASD, and a recent systematic review further highlighted its role in the pathogenesis of ASD [17,18]. In addition, increased NF-κB-related transcripts, activation receptors, and kinases have been reported in patients with schizophrenia [19,20].

The NLRP3 inflammasome, a NOD-like receptor family multiprotein complex, is chiefly localized to microglia and functions as a key mediator of innate immunity in the CNS [21,22]. Activation of the NLRP3 inflammasome downstream of NF-κB signaling leads to caspase-1 activation and, with IL-1β and IL-18 subsequently released as pro-inflammatory cytokines [23,24]. Emerging evidence also supports the involvement of the NLRP3 inflammasome in psychiatric disorders [24,25]. Studies in patients with schizophrenia have demonstrated increased NLRP3 inflammasome expression in peripheral blood mononuclear cells (PBMCs) as well as increased inflammasome protein expression in microglia from postmortem brain tissue [26,27]. In ASD, enhanced NLRP3 inflammasome activation accompanied by increased IL-1β and IL-18 production has also been demonstrated, suggesting a role for NLRP3-mediated innate immune dysregulation in the disorder [28].

Although growing evidence supports the involvement of the TLR4/NF-κB/NLRP3 pathway in several psychiatric disorders, clinical data regarding TLR4, NF-κB, and NLRP3 in ADHD remain limited. Experimental studies in animal models of ADHD have demonstrated increased activation of TLR4, NF-κB, and NLRP3 signaling, accompanied by microglial activation, neuroinflammation, and behavioral abnormalities [29,30,31]. Pharmacological inhibition of this pathway has also been reported to attenuate neuroinflammatory responses and ADHD-like behaviors in experimental models [32,33]. However, the clinical relevance of these experimental findings remains unknown. To the best of our knowledge, no previous clinical study has investigated circulating TLR4, NF-κB, and NLRP3 levels in individuals with ADHD. Therefore, the present study aimed to compare serum TLR4, NF-κB, and NLRP3 levels between children with ADHD and healthy controls and to investigate their associations with ADHD symptom severity.

## 2. Materials and Methods

### 2.1. Study Design and Participants

This single-center case–control study was conducted at the Department of Child and Adolescent Psychiatry, Karadeniz Technical University Faculty of Medicine, Trabzon, Türkiye, with participants enrolled between May and October 2025. The study included 42 children with ADHD and 40 age- and sex-matched healthy controls, all aged 6–12 years. The required sample size was estimated based on a previous study evaluating serum TLR4 levels in children with ASD [15], as comparable clinical data in children with ADHD were not available at the time of study planning. Sample size estimation was conducted a priori based on the TLR4 values reported in that study, with α set at 0.05 and the target power at 90%.

Children with ADHD were required to have a Full-Scale Intelligence Quotient (FSIQ) of ≥80 on the Wechsler Intelligence Scale for Children–Revised (WISC-R). Participants with any comorbid psychiatric disorder, significant medical illness, allergic disease, a history of traumatic brain injury, active infection within the preceding month, psychotropic medication use within the previous three months, or corticosteroid or immunomodulatory treatment within the previous six months were excluded. Healthy controls were additionally required to have no current or lifetime psychiatric disorder and to meet the same exclusion criteria.

All study procedures followed the ethical standards outlined in the Declaration of Helsinki. Participation was entirely voluntary. Written informed consent was obtained from the parents or legal guardians, and verbal assent was obtained from all participating children prior to enrollment. The study was approved by the Ethics Committee of Karadeniz Technical University Faculty of Medicine (date: 28 April 2025, no: 2025/58).

### 2.2. Diagnostic and Symptom Assessment

ADHD diagnoses were established according to the Diagnostic and Statistical Manual of Mental Disorders, Fifth Edition (DSM-5) criteria. Psychiatric evaluations of all children were conducted by a certified child and adolescent psychiatrist using the Turkish version of the Schedule for Affective Disorders and Schizophrenia for School-Age Children–Present and Lifetime Version for DSM-5 (K-SADS-PL-DSM-5-T), which has demonstrated satisfactory validity and reliability in Turkish children [34]. Following the diagnostic assessment, a structured sociodemographic and clinical data form developed by the researchers was completed using information obtained from the children and their parents during the clinical interview.

Symptom severity in ADHD was evaluated with two instruments: the Conners’ Parent Rating Scale–Revised Short Form (CPRS-RS) and the Conners’ Teacher Rating Scale–Revised Short Form (CTRS-RS) [35,36]. Both scales evaluate inattention/cognitive problems, hyperactivity, and oppositional behavior and are widely used for screening and quantifying ADHD symptom severity. Their validity and reliability have been established in Turkish children [37,38]. Parents of children in both the ADHD and control groups completed the CPRS-RS, whereas the CTRS-RS was completed only for children in the ADHD group.

To minimize the potential influence of acute inflammatory conditions on circulating biomarkers, all participants were screened for recent infectious or inflammatory illnesses by systematically inquiring about symptoms including fever, sore throat, cervical swelling, nasal discharge, cough, skin rash, loss of appetite, vomiting, diarrhea, oral ulcers, abdominal pain, and dysuria.

Height and weight were measured for all participants. BMI-for-age percentiles were determined, and weight status was categorized according to the Centers for Disease Control and Prevention (CDC) criteria as underweight (<5th percentile), healthy weight (5th to <85th percentile), overweight (85th to <95th percentile), or obesity (≥95th percentile) [39]. Intellectual functioning was assessed using the Turkish version of the WISC-R, a standardized instrument for assessing cognitive ability in children. The Turkish version of the WISC-R has demonstrated satisfactory validity and reliability [40].

### 2.3. Measurement of Serum TLR4, NF-κB, and NLRP3

All participants underwent venous blood sampling between 08:00 and 10:00 a.m. following an overnight fast, with sample collection carried out under standardized conditions. Before blood collection, participants were instructed to avoid strenuous physical activity and acute stress. Blood specimens were allowed to clot at room temperature before centrifugation, and the separated serum was stored at −80 °C until biochemical analysis. Serum TLR4, NF-κB p65, and NLRP3 concentrations were determined using commercially available ELISA kits (Reed Biotech Ltd., Wuhan, China; Cat. Nos. RE2838H, RE3044H, and RE3548H, respectively) according to the manufacturer’s instructions. According to the manufacturer’s specifications, the detection range and sensitivity were 31.25–2000 pg/mL and 18.75 pg/mL for TLR4, respectively, and 0.16–10 ng/mL and 0.1 ng/mL for both NF-κB p65 and NLRP3, respectively. The manufacturer did not provide separate validation data specifically for pediatric populations. All serum samples were analyzed on the same day by laboratory personnel blinded to the participants’ clinical status. The intra-assay coefficients of variation were 5.23% for TLR4, 7.62% for NF-κB p65, and 7.53% for NLRP3. The standard curves generated during the measurement of TLR4, NF-κB p65, and NLRP3 are provided in Figure S1. Study-specific LOD and LOQ values were not independently determined. None of the measured concentrations fell below the lower limits of the manufacturer-reported assay ranges. Three NF-κB measurements (3/82, 3.7%) exceeded the manufacturer-reported upper assay range.

### 2.4. Statistical Analysis

Statistical analyses were performed using IBM SPSS Statistics for Windows, version 26.0 (IBM Corp., Armonk, NY, USA). Normality of continuous variables was evaluated with the Shapiro–Wilk test. Results for continuous variables are presented as mean ± standard deviation (SD), and categorical variables are summarized as percentages. Depending on the distribution of the data, group comparisons for continuous variables were carried out using either the independent samples *t*-test or the Mann–Whitney U test, whereas the chi-square test was applied to categorical variables. Before the MANCOVA and subsequent ANCOVA analyses, variables with non-normal distributions were transformed using natural logarithms to achieve approximate normality. MANCOVA was performed to evaluate overall group differences across multiple inflammatory biomarkers after adjustment for age, sex, and BMI. Subsequently, separate ANCOVAs were conducted using the same covariates to identify the individual biomarkers contributing to the overall multivariate effect. As a sensitivity analysis, extreme biomarker observations (>3 × IQR beyond the quartiles) were excluded, and the adjusted MANCOVA and ANCOVA analyses were repeated. Correlations between serum biomarker levels and ADHD symptom severity were assessed using Spearman’s rank correlation analysis. To control for the effects of multiple comparisons, the Benjamini–Hochberg false discovery rate (FDR) correction was applied separately to the three unadjusted between-group biomarker comparisons and to the three adjusted biomarker comparisons following ANCOVA. For the correlation analyses, FDR correction was applied separately for each biomarker across the eight Conners scale correlations (four parent-rated and four teacher-rated measures). A two-tailed *p* value < 0.05 was considered statistically significant.

## 3. Results

### 3.1. Demographic and Clinical Characteristics

There were no statistically significant differences between the groups in terms of age (ADHD: 8.17 ± 2.18 years; controls: 8.20 ± 2.03 years; *p* = 0.842), sex distribution (χ^2^ = 0.424, *p* = 0.515) or BMI percentile (*p* = 0.373). Although BMI percentile did not differ significantly between the groups, overweight was more frequent in the ADHD group than in the control group (28.6% vs. 10.0%; χ^2^(1) = 4.499, *p* = 0.034). No participants were classified as underweight or obese. Scores across all CPRS-RS subscales were significantly elevated in the ADHD group (Table 1).

### 3.2. Serum TLR4, NF-κB, and NLRP3 Levels

**Figure 1:**
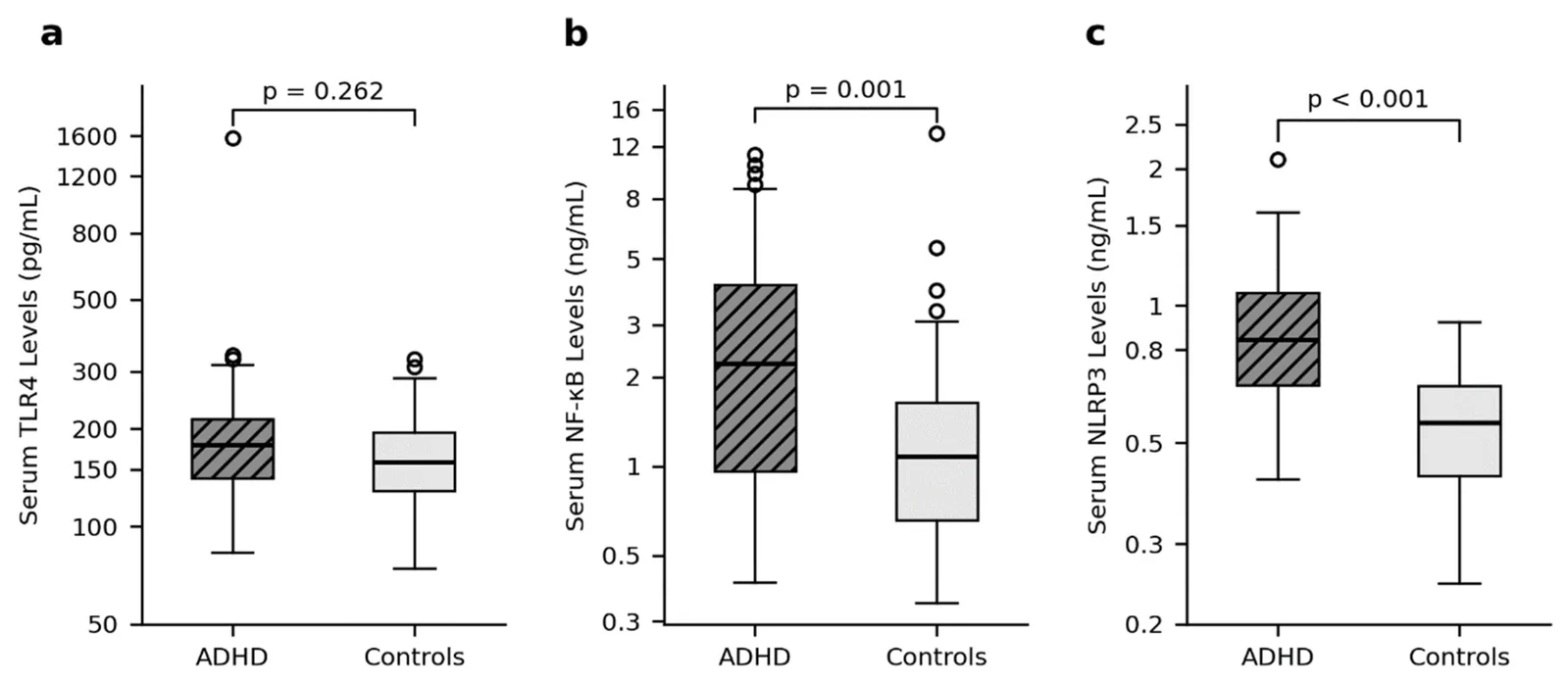
Distribution of serum (**a**) TLR4, (**b**) NF-κB, and (**c**) NLRP3 levels in children with ADHD and healthy controls. The box plots display the median and interquartile range; whiskers indicate values within 1.5 × IQR, and open circles denote outliers. Group differences were assessed using the Mann–Whitney U test.

Serum NF-κB and NLRP3 levels were significantly elevated in children with ADHD compared with healthy controls (z = −3.191, *p* = 0.001 and z = −4.843, *p* < 0.001, respectively), whereas no significant between-group difference was observed in serum TLR4 levels (z = −1.122, *p* = 0.262) (Table 2, Figure 1). After FDR correction, NF-κB and NLRP3 remained significantly higher in the ADHD group (FDR-adjusted *p* = 0.002 and <0.001, respectively), whereas TLR4 remained non-significant (FDR-adjusted *p* = 0.262).

A MANCOVA was conducted to compare inflammatory marker profiles between the ADHD and control groups while controlling for age, sex, and BMI. The analysis revealed a significant overall group effect (Pillai’s Trace = 0.390, F(3,75) = 15.963, *p* < 0.001, ηp^2^ = 0.390). To account for potential confounding effects of age, sex, and BMI, ANCOVA was performed using log-transformed biomarker values. After adjustment for age, sex, and BMI, the findings remained essentially unchanged. Children with ADHD continued to show significantly higher ln-NF-κB [F(1,77) = 16.663, *p* < 0.001, η^2^p = 0.178] and ln-NLRP3 levels [F(1,77) = 30.647, *p* < 0.001, η^2^p = 0.285] than healthy controls. In contrast, no significant between-group difference was observed for ln-TLR4 levels [F(1,77) = 1.596, *p* = 0.210, η^2^p = 0.020] (Table 3). These findings remained unchanged after FDR correction across the three biomarker comparisons (FDR-adjusted *p* < 0.001 for NF-κB and NLRP3 and *p* = 0.210 for TLR4). In the sensitivity analysis, three extreme observations (one for TLR4 and two for NF-κB) were excluded. The adjusted multivariate group effect remained significant (Pillai’s Trace = 0.444, F(3,72) = 19.203, *p* < 0.001). In the corresponding ANCOVA analyses, TLR4 remained non-significant (*p* = 0.316), whereas NF-κB and NLRP3 remained significant (both *p* < 0.001). In an additional sensitivity analysis excluding all three NF-κB observations exceeding the manufacturer-reported upper assay range, the adjusted between-group difference in NF-κB remained significant (F = 21.62, *p* < 0.001, partial η^2^ = 0.226).

### 3.3. Correlations Between Serum TLR4, NF-κB, and NLRP3 Levels and ADHD Symptom Severity

Correlation analyses revealed no significant associations between serum TLR4, NF-κB, or NLRP3 levels and parent-rated CPRS-RS scores (all *p* > 0.05). Regarding teacher-rated CTRS-RS scores, NF-κB levels showed a positive correlation with the Hyperactivity subscale (r = 0.430, *p* = 0.004) and this association remained significant after FDR correction (adjusted *p* = 0.032). No other significant correlations were detected between biomarker levels and teacher-rated symptom measures (Table 4).

## 4. Discussion

To our knowledge, this is the first clinical study to evaluate serum TLR4, NF-κB, and NLRP3 levels in children with ADHD. The principal findings of the present study were that children with ADHD exhibited significantly higher serum NF-κB and NLRP3 levels than healthy controls, whereas serum TLR4 levels did not differ between groups. Furthermore, serum NF-κB levels were positively associated with teacher-rated hyperactivity symptoms, while no significant associations were observed for TLR4 or NLRP3. Together, these findings provide preliminary evidence of differential alterations in these inflammatory markers in children with ADHD, rather than uniform changes across all components of the TLR4/NF-κB/NLRP3 pathway.

In the present study, serum TLR4 levels did not differ significantly between children with ADHD and healthy controls. This finding appears to differ from experimental studies in animal models of ADHD, which have consistently demonstrated activation of the TLR4/NF-κB/NLRP3 signaling pathway together with enhanced neuroinflammation and behavioral abnormalities [29,30,32,33]. However, evidence regarding TLR4 alterations in children with ADHD remains scarce, making the interpretation of our findings challenging. Given the limited clinical evidence in ADHD, findings from other neurodevelopmental disorders may provide useful context for interpreting the present results.

In this context, studies in ASD provide relevant comparative evidence. Nadeem et al. reported increased TLR4 expression in peripheral T lymphocytes from children with ASD [41], while Al-Harbi et al. observed enhanced TLR4/NF-κB signaling in B lymphocytes [14]. More recently, Kiyat et al. reported significantly higher circulating soluble TLR4 levels in children with ASD than in healthy controls, with serum TLR4 levels showing a positive correlation with hyperactivity symptoms [15]. One proposed explanation for these findings involves alterations of the gut–brain axis. Increased intestinal permeability may facilitate the translocation of bacterial lipopolysaccharide (LPS), a major ligand of TLR4, into the systemic circulation, thereby promoting persistent TLR4-mediated inflammatory signaling [41,42]. Although disturbances of the gut–brain axis have also been suggested in ADHD, the available evidence appears to be less consistent than that reported for ASD [43]. This difference in the available evidence may provide one possible context for understanding the discrepant findings regarding circulating TLR4 levels between ASD and ADHD.

Schizophrenia findings may further inform our results, as it shares neuroinflammatory and cognitive deficits with ADHD, and TLR4 dysregulation is implicated in its pathophysiology [44]. Previous studies in schizophrenia have reported inconsistent alterations in TLR4 expression and activity, including increased, decreased, or unchanged levels, as well as variable associations with cognitive performance [45,46,47,48]. These findings suggest that TLR4 may have complex regulatory roles beyond the initiation of inflammatory responses, including processes related to neuroplasticity and cognition [45,49]. One possible explanation for these inconsistencies involves compensatory regulatory mechanisms within TLR4 signaling. Downstream inflammatory mediators, including NF-κB and IL-1β, may remain activated despite reduced TLR4 expression [47], while endogenous negative regulators induced following TLR activation may limit excessive inflammatory signaling [50]. Such regulatory mechanisms may provide a possible context for our finding of unchanged serum TLR4 levels alongside elevated NF-κB and NLRP3 levels. Taken together, these observations suggest that circulating TLR4 levels alone may not fully reflect the broader inflammatory signaling processes in ADHD.

Serum NF-κB levels were significantly elevated in the ADHD group relative to healthy controls. The elevated NF-κB levels observed in our study are consistent with findings from experimental ADHD models, in which activation of NF-κB signaling has been associated with enhanced neuroinflammation and ADHD-like behavioral abnormalities [29,30,32,33]. Clinical studies in other neuropsychiatric conditions also provide supporting context. Increased NF-κB levels and related inflammatory alterations have been reported in children with ASD [18,51], while altered NF-κB-related gene expression has also been observed in schizophrenia [44]. Collectively, these findings support the relevance of NF-κB-related inflammatory signaling across different neuropsychiatric conditions.

Notably, serum NF-κB levels were elevated despite the absence of a significant difference in circulating TLR4 levels. This finding is biologically plausible because NF-κB activation is regulated through multiple upstream signaling pathways and is not exclusively dependent on TLR4 activation [10]. Accordingly, alterations in TLR4 and NF-κB do not necessarily occur in parallel. For example, MacDowell et al. reported increased TLR4 expression in the cerebellum of patients with chronic schizophrenia despite unchanged NF-κB levels and reduced NF-κB functional activity [52]. Thus, the higher serum NF-κB levels observed in our study cannot be attributed specifically to TLR4-mediated signaling and may reflect alterations involving other upstream mechanisms. Further studies examining these mechanisms are needed to clarify the significance of elevated NF-κB levels in ADHD.

Children with ADHD also showed significantly higher serum NLRP3 levels than healthy controls. This observation is in line with experimental ADHD research demonstrating increased NLRP3 expression in association with TLR4/NF-κB/NLRP3 signaling [29,32,33]. Szabó et al. reported increased NLRP3 expression and caspase-1 activity in peripheral blood mononuclear cells from individuals with ASD, together with increased inflammatory cytokine secretion, while pharmacological inhibition of NLRP3 attenuated these responses [28]. Experimental studies have similarly shown that suppression of NLRP3 activation is associated with improvements in neurobehavioral abnormalities [31]. However, whether these experimental observations have any therapeutic relevance to ADHD remains unknown and requires further investigation.

Evidence from schizophrenia research may also help contextualize our findings. Liu et al. reported no significant between-group difference in NLRP3 mRNA expression among first-episode, drug-naïve patients compared with healthy controls, although an NLRP3 polymorphism was associated with cognitive performance in that sample [53]. In contrast, Ozdamar Unal et al. demonstrated higher IL-1β, IL-18, and NLRP3 gene expression in PBMCs of patients with schizophrenia compared with healthy controls. Higher expression of NLRP3 and IL-18 was associated with poorer functioning, while IL-18 expression was also associated with greater positive symptom severity [26]. These findings highlight the heterogeneity of NLRP3-related alterations across clinical populations.

Taken together, these findings provide context for the elevated serum NLRP3 levels observed in children with ADHD. Furthermore, the concurrent elevation of serum NF-κB and NLRP3 levels despite unchanged circulating TLR4 concentrations suggests that these inflammatory markers may be differentially altered rather than reflecting uniform activation of the TLR4/NF-κB/NLRP3 pathway. Importantly, circulating NLRP3 concentrations do not directly reflect inflammasome activity within the central nervous system. Further studies integrating peripheral biomarkers with functional and mechanistic assessments are needed to clarify the significance of NLRP3 alterations in ADHD.

In addition to these mechanistic considerations, potential metabolic, behavioral, and psychosocial influences should be considered when interpreting the observed inflammatory differences. Overweight was more frequent among children with ADHD in our sample, in line with previous meta-analytic evidence of an association between ADHD and overweight/obesity in children and adolescents [54]. This difference is relevant to the interpretation of the inflammatory findings, as excess adiposity and related metabolic alterations are associated with chronic low-grade inflammation involving NLRP3, TLR4-related signaling, and NF-κB pathways [55,56,57]. Dietary and physical activity patterns may also influence peripheral inflammatory profiles [58,59], while sleep characteristics and psychosocial stress may represent additional sources of variability [60,61]. Importantly, the between-group differences in NF-κB and NLRP3 remained significant after adjustment for BMI. Nevertheless, BMI alone may not fully capture adiposity or metabolic status, and detailed metabolic parameters, dietary patterns, habitual physical activity, sleep characteristics, and psychosocial stress were not systematically assessed. Therefore, residual confounding cannot be excluded, and the potential contribution of these factors should be considered when interpreting the observed between-group differences in inflammatory markers. Future studies incorporating more comprehensive assessments of metabolic status, body composition, lifestyle factors, sleep, and psychosocial stress may help clarify their contribution to these findings.

Regarding symptom severity, serum TLR4 and NLRP3 levels were not significantly correlated with either parent- or teacher-rated ADHD symptoms. In contrast, serum NF-κB levels showed a moderate positive correlation with teacher-rated hyperactivity scores. However, this association was isolated and was not observed across parent ratings or other teacher-rated symptom domains; therefore, it should be interpreted cautiously rather than as evidence of a consistent relationship between NF-κB levels and ADHD symptom severity. Experimental studies have reported associations between NF-κB activation and behavioral abnormalities in ADHD models [29,32,33,62,63], while Kiyat et al. reported an association between circulating TLR4 levels and hyperactivity symptoms in children with ASD [15]. However, these findings provide only indirect context for the association observed in our study. Given the limited number of significant correlations and the cross-sectional design, the observed NF-κB–hyperactivity association should be considered exploratory and requires confirmation in larger longitudinal studies.

The present study has several strengths. To our knowledge, this study represents the first clinical investigation of serum TLR4, NF-κB, and NLRP3 levels in children with ADHD. Another important strength of the present study is the inclusion of a carefully characterized ADHD sample that was free from psychotropic medication for at least 3 months prior to enrollment and had no psychiatric comorbidities. Previous studies have suggested that psychotropic medications may influence inflammatory biomarkers, potentially confounding the interpretation of peripheral immune parameters [5,64]. Likewise, inflammatory alterations have been reported not only in ADHD but also in several psychiatric disorders commonly comorbid with ADHD, including depressive and anxiety disorders [12,13,65]. Therefore, the exclusion of these potential confounding factors contributed to a more homogeneous clinical sample and facilitated the interpretation of the observed biomarker differences.

The study has several limitations. First, the cross-sectional nature of the study does not allow the direction of the relationship between inflammatory alterations and ADHD to be determined. Accordingly, the present data cannot clarify whether the observed elevations in NF-κB and NLRP3 precede the onset of ADHD, arise as a consequence of the disorder or associated factors (e.g., chronic stress or sleep disturbance), or represent an epiphenomenon unrelated to core ADHD pathophysiology. Second, although the inclusion of children who had been free from psychotropic medication for at least 3 months and had no psychiatric comorbidities reduced important sources of confounding, it may limit the generalizability of our findings to the broader ADHD population. Third, the relatively modest sample size may limit the precision and stability of the estimates, particularly given the substantial variability in some biomarker levels, and may increase the sensitivity of the findings to individual observations. Although sensitivity analyses excluding extreme observations yielded results consistent with the primary analyses, these findings should be interpreted cautiously in light of the sample size and biomarker variability. Furthermore, although FDR correction was applied to address multiple testing, the possibility of type I error cannot be entirely excluded, while the modest sample size may also increase the risk of type II error, particularly for smaller effects. In addition, the single-center design and absence of an independent replication cohort limit the generalizability of the findings. Potentially relevant inflammatory confounders, including metabolic parameters, dietary and physical activity patterns, sleep characteristics, and psychosocial stress, were not systematically assessed and therefore could not be controlled for. Although BMI was included as a covariate, residual confounding related to these factors cannot be excluded. In addition, study-specific LOD and LOQ values were not independently determined for the ELISA assays, which limits the complete analytical characterization of the biomarker measurements. Finally, only peripheral serum concentrations of TLR4, NF-κB, and NLRP3 were evaluated. Although peripheral biomarkers provide valuable information regarding systemic inflammatory processes, they cannot be assumed to directly reflect inflammatory processes within the central nervous system. Moreover, because other inflammatory markers such as IL-1β, IL-6, TNF-α, and CRP were not concurrently assessed, the present study cannot determine whether the observed alterations are specific to the investigated TLR4/NF-κB/NLRP3 components or reflect a broader peripheral inflammatory state. In addition, we measured circulating concentrations of these molecules but did not evaluate downstream inflammatory mediators or functional activation of the TLR4/NF-κB/NLRP3 signaling pathway. Accordingly, these findings should be considered preliminary and hypothesis-generating. Future multicenter longitudinal studies integrating peripheral biomarkers with complementary mechanistic approaches are warranted to further clarify the relevance of these inflammatory alterations in ADHD.

## 5. Conclusions

We found significantly higher serum NF-κB and NLRP3 levels, but not TLR4 levels, in children with ADHD compared with healthy controls. These findings provide preliminary clinical evidence of alterations in selected inflammatory markers in children with ADHD. The concurrent elevation of NF-κB and NLRP3 despite unchanged TLR4 levels suggests that these markers may be differentially altered rather than reflecting uniform activation of the TLR4/NF-κB/NLRP3 pathway. Given the exploratory nature of the study, these findings should be considered hypothesis-generating and require independent replication. Future longitudinal and multicenter studies integrating peripheral biomarkers with functional and mechanistic assessments are needed to clarify the relevance of these inflammatory alterations to ADHD pathophysiology.

## Figures and Tables

**Table 1:** Demographic and clinical characteristics of the ADHD and control groups.

		ADHD Mean ± SD/ *n* (%)	Control Mean ± SD *n* (%)	z/χ^2^	*p*
Age (years)		8.17 ± 2.18	8.20 ± 2.03	−0.199 ^a^	0.842
Gender	Female	8(19)	10(25)		
	Male	34(81)	30(75)	0.424 ^b^	0.515
BMI percentile		61.05 ± 26.88	57.55 ± 23.24	0.891 ^a^	0.373
Weight status					
Healthy weight		30 (71.4)	36 (90.0)		
Overweight		12 (28.6)	4 (10.0)	4.499 ^b^	**0.034**
CPRS-RS					
Oppositional Tendencies		9.71 ± 4.16	3.37 ± 2.47	−6.344 ^a^	**<0.001**
Cognitive Problems/Inattention		11.40 ± 4.65	2.25 ± 2.52	−6.993 ^a^	**<0.001**
Hyperactivity		10.38 ± 4.19	1.75 ± 1.67	−7.590 ^a^	**<0.001**
ADHD Index		21.40 ± 5.89	4.92 ± 3.50	−7.644 ^a^	**<0.001**
CTRS-RS					
Oppositional Tendencies		6.04 ± 4.36			
Cognitive Problems/Inattention		5.36 ± 4.02			
Hyperactivity		13.64 ± 5.05			
ADHD Index		23.00 ± 5.78			

^a^ Mann–Whitney U test; ^b^ Chi-Square test; Bold values indicate statistical significance (*p* < 0.05).

**Table 2:** Comparison of serum TLR4, NF-κB and NLRP3 levels of the ADHD and control groups.

	ADHD (*n*:42)	Control (*n*:40)	Mann–Whitney U Test	
	Mean ± SD	Mean ± SD	z	*p*
**TLR4 (pg/mL)**	215.26 ± 224.52	169.41 ± 58.41	−1.122	0.262
**NF-κB (ng/mL)**	3.33 ± 2.97	1.65 ± 2.18	−3.191	**0.001**
**NLRP3 (ng/mL)**	0.90 ± 0.37	0.56 ± 0.17	−4.843	**<0.001**

Bold values indicate statistical significance (*p* < 0.05).

**Table 3:** Comparison of log-transformed serum TLR4, NF-κB, and NLRP3 levels between patients with ADHD and controls using ANCOVA.

	ADHD (*n*:42)	Control (*n*:40)	ANCOVA			
	Mean ± SD	Mean ± SD	F	*p*	η^2^p	Adjusted Difference (95% CI)
**ln-TLR4 (pg/mL)**	5.19 ± 0.49	5.08 ± 0.34	1.596	0.210	0.020	0.117 (−0.068 to 0.303)
**ln-NFκB (ng/mL)**	0.80 ± 0.95	0.11 ± 0.80	16.663	<0.001	0.178	0.662 (0.339 to 0.985)
**ln-NLRP3 (ng/mL)**	−0.18 ± 0.40	−0.62 ± 0.32	30.647	<0.001	0.285	0.445 (0.285 to 0.606)

**Table 4:** Correlations between TLR4, NF-κB, and NLRP3 levels and Conners scale scores in the ADHD group.

	TLR4		NF-κB		NLRP3	
	r	*p*	r	*p*	r	*p*
CPRS-RS						
Oppositional Tendencies	−0.232	0.139	0.086	0.588	0.028	0.861
Cognitive Problems/Inattention	−0.028	0.861	−0.150	0.344	0.032	0.838
Hyperactivity	−0.091	0.566	0.183	0.247	0.001	0.993
ADHD Index	−0.021	0.897	−0.121	0.446	−0.083	0.601
CTRS-RS						
Oppositional Tendencies	0.091	0.566	−0.054	0.735	−0.006	0.968
Cognitive Problems/Inattention	0.088	0.578	−0.007	0.965	−0.258	0.099
Hyperactivity	−0.012	0.939	0.430	0.004 *	0.016	0.921
ADHD Index	0.005	0.977	0.164	0.300	−0.124	0.433

* *p* < 0.05; Spearman’s rho correlation test.

## Data Availability

The datasets generated and/or analyzed during the current study are available from the corresponding author on reasonable request. The data are not publicly available due to privacy restrictions and protection of patient confidentiality.

## Supplementary Materials

The following supporting information can be downloaded at: https://www.mdpi.com/article/10.3390/children13091278/s1, Figure S1. Standard curves used for the ELISA measurements of (A) TLR4, (B) NF-κB p65, and (C) NLRP3.

## Abbreviations

ADHD	Attention-deficit/hyperactivity disorder
TLR4	Toll-like receptor 4
NF-κB	Nuclear factor-kappa B
NLRP3	NOD-like receptor family pyrin domain containing 3
ELISA	Enzyme-linked immunosorbent assay
BMI	Body mass index
ASD	Autism spectrum disorder
DSM-5	Diagnostic and Statistical Manual of Mental Disorders, Fifth Edition
K-SADS-PL	Schedule for Affective Disorders and Schizophrenia for School-Age Children–Present and Lifetime Version
WISC-R	Wechsler Intelligence Scale for Children–Revised
FSIQ	Full-Scale Intelligence Quotient
CPRS-RS	Conners’ Parent Rating Scale–Revised Short Form
CTRS-RS	Conners’ Teacher Rating Scale–Revised Short Form
SD	Standard deviation
MANCOVA	Multivariate analysis of covariance
ANCOVA	Analysis of covariance
TNF-α	Tumor necrosis factor-alpha
IL	Interleukin
PBMCs	Peripheral blood mononuclear cells
LPS	Lipopolysaccharide
PRRs	Pattern-recognition receptors
