# Supplementary material for: Serum TLR4, NF-κB, and NLRP3 Levels in Children with Attention-Deficit/Hyperactivity Disorder: A Case–Control Study
Source: Children (Basel). 2026 Sep 21;13(9):1278. doi: 10.3390/children13091278 (PMC13605406; doi:10.3390/children13091278)
Supplement: Supplementary file 1 [file children-13-01278-s001.zip › children-4519839-supplementary.pdf]

Figure S1

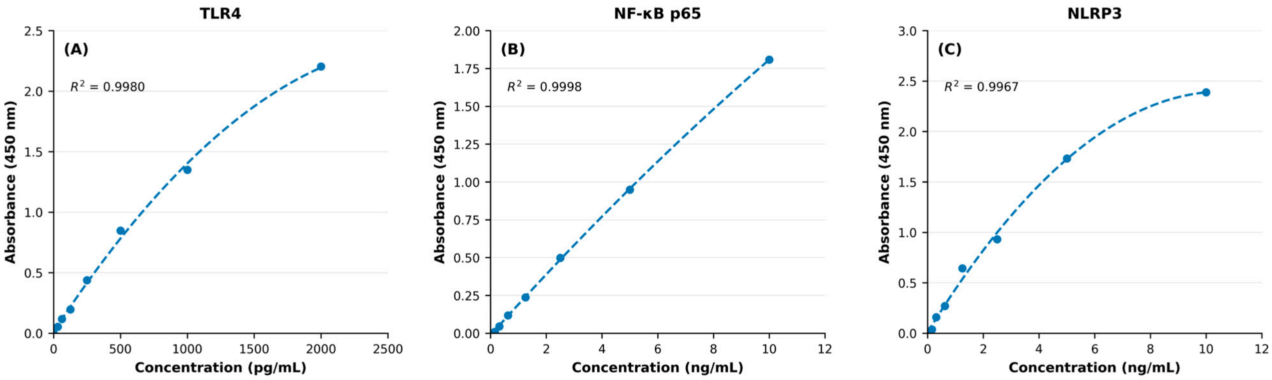

Figure S1. Standard curves used for the ELISA measurements of (A) TLR4, (B) NF-κB p65, and (C) NLRP3.
